# Using action research and a community-academic partnership to understand clinical trial participation: a patient-centered perspective

**DOI:** 10.1186/s40900-024-00593-z

**Published:** 2024-06-13

**Authors:** Sara Santarossa, Michele Baber, Janine Hussein, Chrystal Oley, Kristen Slangerup, Dana Murphy, Karen E. Kippen

**Affiliations:** 1 Henry Ford Health System, Department of Public Health Sciences, Patient-Engaged Research Center, Detroit, MI USA; 2 AstraZeneca, Inc, 1 MedImmune Way, Gaithersburg, MD USA

**Keywords:** Action research, Community-academic partnership (CAP), Patient-centered, clinical trials

## Abstract

**Background:**

Clinical trials that are patient-centered appear to be more successful (e.g., clinical outcomes, improved communication, mutual empowerment, changed attitudes), thus, action research may be a field of importance. The current study explores the Formation and Execution of Activities phases of a community-academic partnership (CAP).

**Methods:**

Members consisted of industry stakeholders, a healthcare/academic institution, and patients/families with lived experiences as cancer survivors and/or caregivers. Retrospectively, CAP members described the facilitating and/or hindering factors present in the partnership development. A document review process was used. Field notes from three CAP meetings, which focused on understanding clinical trial participation, were analyzed using a thematic approach.

**Results:**

Seven facilitating and three hindering factors were present. Interpersonal (vs. operational) processes were referenced as influential facilitating factors more often. Themes that emerged included ‘trials as a treatment option’, ‘leaving a legacy’, and ‘timing is critical.’

**Conclusion:**

This study provides a patient-centered perspective on barriers/challenges of clinical trial participation and how to improve future perceptions.

## Abstract (plain language summary)

Clinical trials are more successful when patients are engaged, and their perspectives have been considered in the study design. Community-academic partnerships (CAPs) are one way to ensure patients are more engaged in the research process by creating a collaboration where all parties involved play an equitable role. We provide an example of a CAP with an industry stakeholder, a healthcare/academic institution, and patients as well as families with lived experiences as cancer survivors and/or caregivers. Described here two phases of the CAP: the Formation and the Execution of Activities phases. The Formation phase covers the collaboration process and development of the CAP. In our study, to better understand this phase, CAP members described what did and did not go well during the partnership development. We found more aspects went well than did not and that processes related to the quality of the relationship and communication among CAP members were important. The Execution of Activities phase focuses on how the CAP is working towards an agreed upon outcome. In our study, to better understand this phase, we reviewed notes taken at previous CAP meetings that focused on exploring participation in clinical trials as a treatment option. We found that when it comes to participation in clinical trials, patients and/or caregivers with lived experiences with cancer, felt that timing of this treatment option was important and that reasons for participation included wanting to leave a legacy. In this paper we describe some challenges of clinical trial participation, identified by patients and caregivers, and discuss how to improve views of clinical trial participation in the future.

## Background

Clinical trials are essential to the development of new and innovative medicine. Literature suggests that despite laborious efforts, many studies face numerous challenges when it comes to recruitment and retention in clinical trial [1–3]. Moreover, it is important that clinical trials represent the patient populations that will be treated with new medicines, yet across industry there is a lack of diverse racial/ethnic representation. Recent literature suggests, across all therapeutic areas only 9% of trial participants were Black or African American and only 5% in oncology studies [4]. Constructing a better understanding of patient-facing obstacles, particularly among patients of diverse racial/ethnic backgrounds, is thus imperative to recognize what facilitates or hinders participation in clinical trials as a treatment option. Research methods that support higher levels of patient engagement and study designs that are patient-centered have been found to yield more successful clinical trial (e.g., clinical outcomes, improved communication, mutual empowerment, changed attitudes) [5–7]. Limited research has explored explicitly how collaborative, patient-centered efforts involving multiple stakeholders (e.g., scholars, industry stakeholder, patients) can enable the flow of research knowledge about lack of clinical trial participation and address the best practices in how to improve it. A promising component in the development of patient-centered clinical trial designs is the use of action research.

Action research, an umbrella term, has been described as “a family of practices of living inquiry that aims, in a great variety of ways, to link practice and ideas in the service of human flourishing,” (p.1) where the orientation of change is with others [8]. Community-academic partnerships (CAPs), which are within the practices of action research, are designed to increase collaborative efforts between researchers and the community. CAPs have been defined as partnerships in which researchers and community stakeholders have equitable control in addressing a cause that is primarily relevant to the community of interest and aims to achieve a goal relevant to both community members (representatives or agencies) and researchers [9]. Based on a systematic review, of primarily studies that took place in the United States (*n* = 19/50 studies outside of the United States), CAPs are multi-directional, addressing the needs for improved collaboration between academics and community practitioners as well as hoping to disseminate and implement promising interventions and community programs [9]. CAPs are believed to increase the effectiveness and feasibility of action research [9, 10].

The theory-based Model of Research-Community Partnerships [10], suggests three phases (i.e., Formation, Execution of Activities, and Sustainment) that illustrate the iterative processes of a CAP’s development and conceptualize outcome constructs of these efforts. There are important processes that correspond to each phase: [1] Formation, corresponds with the collaboration process and development of the CAP (i.e., Interpersonal and Operational Processes) and subsequent facilitating and hindering factors [2], Execution of Activities, includes proximal (process) outcomes (e.g., partnership synergy, knowledge exchange, and the creation of tangible projects) focusing on the partnership functioning of the CAP, and [3] Sustainment, focuses on distal outcomes (e.g., policy changes) [10].

CAPs have been utilized in such fields as health and medicine, encompassing topics on social services as well as interventions for medical conditions [5–7, 11]. This type of action research can enable high-caliber care and improvements in quality of life for patients [5–7], however, researchers are hesitant to implement this type of methodology, due to limited training with CAPs, increased time commitment, and institutional pressure for funding and faster research outcomes [10]. Moreover, in a systematic review of the state of CAPs literature, it was reported that most CAP research studies do not describe important membership characteristics such as initiation, the number of partners, the duration of the partnership, or the funding sources [9]. In addition, most CAP research has used a cross-sectional design and has not compared the collaborative process at multiple timepoints [9]. Therefore, although the overall aim of the current study was to use a CAP to better understand what facilitates or hinders participation in clinical trials as a treatment option, the authors felt it was important to fully explore the nature of the CAP. The collaborating partners of the CAP outlined in the current study include an industry stakeholder, a healthcare/academic institution, and groups of patients and caregivers. While being guided by the Model of Research-Community Partnership [10], using outcome constructs of the Formation and the Execution of Activities phases, the purpose of the present study included:

1. To systematically report the CAP’s characteristics, highlighting the longitudinal relationships, and describing the interpersonal and operational processes that have facilitated and/or hindered this collaborative effort (*Formation Phase*).
2. Through a series of informal meetings, use the CAP’s effort to address barriers and challenges of clinical trial participation as an accepted treatment option, specifically improving the perception of clinical trial participation from a patient-perspective (*Execution of Activities Phase*).

## Method

The current study uses the Model of Research-Community Partnership [10] to assist in systematically reporting the characteristics of the CAP (*Formation Phase*) as well as to interpret outcomes of the partnership effort during the *Execution of Activities Phase*. As the distal outcomes of the CAP is ongoing and have not yet been measured, the third phase *Sustainment*, is not evaluated nor presented in the current study. The CAP was comprised of an industry stakeholder (i.e., AstraZeneca; a pharmaceutical and biopharmaceutical company), a healthcare/academic institution (i.e., Henry Ford Health, Detroit, MI; specifically, Henry Ford Health’s Patient-Engaged Research Center; PERC), and patients as well as families with lived experiences as cancer survivors and/or caregivers (i.e., PERC’s Patient Advisors; PAs, and subsequently Patient Family Advisory Council; PFAC). A document review process [12] was used as a data collection method for evaluation in the current study. Document review enabled the authors to gather background information and needed data to answer evaluation question pertaining to the CAP. The document review process involved the analysis of field notes, from both Henry Ford Health (HFH) and AstraZeneca, taken during the three partnership synergy and knowledge exchange meetings.

### Systematically reporting the CAP’s characteristics: formation phase

#### Collaborative process

HFH provides both health care services (acute, specialty, primary preventive care) and health insurance to a diverse population in Michigan (e.g., 9% Hispanic, 25% non-Hispanic Black). AstraZeneca has a portfolio of products for major disease areas including cancer, cardiovascular, gastrointestinal, infection, neuroscience, respiratory and inflammation. HFH and AstraZeneca have a historical relationship, spanning many years, interacting with numerous disciplines and divisions within HFH including Pharmacy, Public Health Sciences, and the Clinical Trials Office. This study reflects the first time, however, that the collaborative relationship between HFH and AstraZeneca is framed as a CAP and evaluated using the Model of Research-Community Partnership [10]. Moreover, for the purpose of this article, the authors will focus on AstraZeneca’s working relationship (i.e., CAP) with HFH’s PERC, PAs, and their PFACs.

PERC’s decision to initiate a relationship with AstraZeneca was based on the positive relationships HFH had already built with this industry stakeholder. Historical context of PERC and the development of their high functioning Flexible Engagement Model to recruit, engage and retain over 480 PAs (~ 34% self-report as African American) as partners in clinical care pathway improvement and funded research projects, is reported in detail elsewhere [13]. Briefly, PAs are trained by PERC to effectively engage with health system leaders, healthcare providers, health plan administrators, and other key community and industry stakeholders addressing a variety of topics such as improving patient care policies, processes, communication materials, quality improvement activities, and safety efforts, important to patients and families. Notably, PAs do not have to be patients of HFH, allowing for broader perspectives.

Many PAs are members on PFACs (*N* = 14 throughout HFH). Typically, a PFAC consist of 15–20 PAs who “collectively drive meeting agendas by identifying priorities and topics they would like to focus on through a standardized strategic planning process” (p.38) [13]. PAs, after the onboarding and training process, are allowed to request to join PFACs that are open for recruitment. PERC does not turn anyone away from joining as a PA, however, they would not be able to join a PFAC unless their experience align (e.g., a PA wanting to join the Cancer Center PFAC would be required to have experience as cancer patient/survivor/caregiver).

**Fig. 1:**
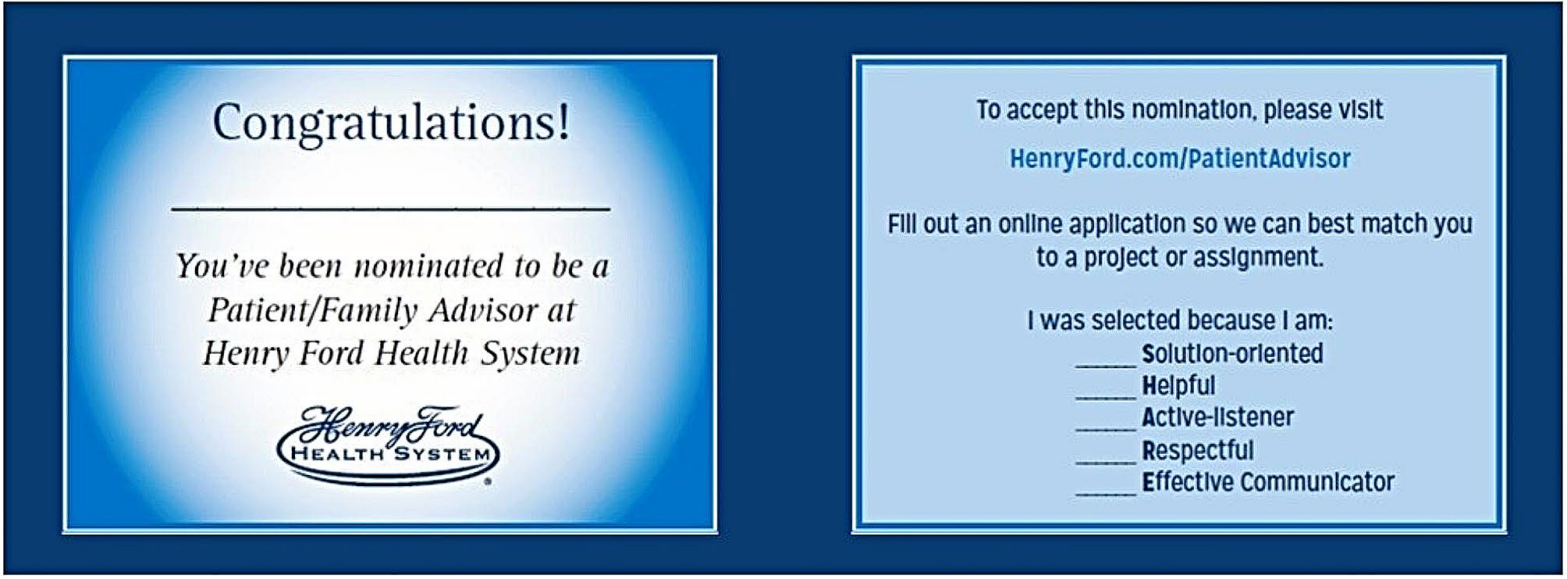
The Nomination card process utilized for recruitment of patient advisors; front on left and backside on right

In 2015, several PFACs that were related to cancer care began to develop. Of importance to the current study are the Cancer Center PFAC and the Head and Neck Cancer PFAC. The Cancer Center PFAC was created in response to a request from the CEO of HFH’s flagship hospital in Detroit, MI. HFH was developing plans to build a destination cancer center and wanted to have patients and families involved from the beginning in all phases of its development including the architecture and design, ancillary care programs, as well as research and clinical care pathway improvement. Clinical care pathways standardize care for patients with a similar diagnosis, procedure, or symptom. This is used as one example of the meaningful work that Cancer Center PFAC were engaged in. For example, clinical providers and clinical pathway owners wanted input from the patient advisors on the Cancer Center PFAC to inform the providers if the patients perceived they were receiving the right care services at the right time during their cancer treatment and survivorship journey. The feedback included the timing of services and if additional ancillary services would better support the cancer patients and their caregivers. PERC’s Nomination Card Process (see Fig. 1 for an image of the Nomination Card) was utilized as one form of recruitment of PAs. Details for the Nomination Card Process are published elsewhere, but briefly, the nomination card, provided by a HFH staff, refer patients/caregivers to the PERC website where they can fill out an application form for the PA Program [13]. Through this process, approximately 20 PA applicants were contacted, completed a phone screening, and attended an in-person orientation. Physicians led outreach for participation in the Head and Neck Cancer PFAC, sending personal letters to their patients asking them to participate. PERC personnel followed up with a phone call and an in-person orientation.

Due to PERC’s continual pursuit of clinical and quality improvement as well as research funding opportunities, a project aiming to ‘Engage Patients and Other Stakeholders to Develop a Patient-Centered Research Agenda for Cancer Precision Medicine’ was awarded two years of funding (2017–2019) from the Patient Centered Outcomes Research Institute’s (PCORI). This project engaged a broad group of HFH stakeholders including providers, health plan CEOs, researchers; PAs including the Cancer Center and Head and Neck Cancer PFAC; and industry stakeholders including Syapse (i.e., IT company focused on precision medicine in oncology) and AstraZeneca. Tangible outcomes of this PCORI funded project included: hosting a Precision Medicine Symposium (2017), a Precision Medicine Research Agenda, as well as videos, a white paper and a dissemination plan regarding the group’s work.

Over the tenure of PCORI funding, AstraZeneca personnel built trusting relationships across the HFH stakeholder groups. Specifically, the PAs had come to know them by name and understood the expertise and scientific resources of the pharmaceutical company through their in-person attendance at bi-monthly/quarterly meetings, their presentations at the symposium, and their willingness to answer questions and actively listen to PAs’ stories and ideas. There was an attempt to provide a mutual benefit for all partners and AstraZeneca representatives attended the annual PERC Patient Advisor Retreats, events that both educate and build new communication skills for PAs. After the conclusion of the PCORI funding, PERC and AstraZeneca personnel continued to meet and look for ways to move the research agenda forward and engage the Cancer Center as well as the Head and Neck Cancer PFAC through other opportunities. One such opportunity was related to understanding patient and family perceptions related to participation in clinical trials, thus the focus of this article.

Importantly, at the time of this article, the Cancer Center and Head and Neck Cancer PFAC consisted of a total of 24 PAs where 21% were African American and majority identified as women (54%). In addition, a normal distribution of age groups was represented, ranging from 35 to 75 + years of age, with the 65–74 years age range being the most prominent at 29%. Seventeen of the PAs identified as patients, six identified as caregivers, and one identified as both a patient and caregiver. Not all PAs actively attended all meetings, however, meeting attendance is captured in Table 1.

### **Using the cap effort to address proximal outcomes: execution of activities phase**

#### Partnership synergy and knowledge exchange

PFAC meetings were structured to have a focused discussion with prompts centered on different areas pertaining to the objective of this CAP effort and research purpose (e.g., what facilitates or hinders participation in clinical trials as a treatment option, among a diverse patient population). Three meetings were held over a three-month period with the first two meetings focused on facilitating discussion, and the third meeting focused on how tangible actions could be developed. Details of meeting structure, including participant breakdown, are outlined in Table 1.

**Table 1:** Partnership synergy and knowledge exchange detailed meeting structure

	Participants	Discussion prompts/Focus	Insights AstraZeneca aimed to gain	Insights patients/caregivers aimed to gain
Meeting 1	*N* = 15 *n* = 8 PAs (5 cancer survivors, 3 caregivers) *n* = 4 PERC personnel *n* = 3 AstraZeneca personnel	What is your impression of clinical trial participation? Where did that come from? Did you consider research as a “good” option? Would you recommend a clinical trial to a friend or family member?	General: What are patients’ impressions of clinical research? Specific: What are the important details of a clinical trial that would increase the likelihood participation in a specific protocol?	General (i.e., any project a PA works on): • Helping future patients • Being a voice for patients/caregivers • Feeling like valued partners Specific: • Wanting more education on clinical trials based on their lack of knowledge during their own cancer journey. • Wanting future patients/caregivers to be presented with all options up front to determine the best course of treatment for them. • How and when information is presented to patients/caregivers (why education for clinicians is the first step)
Meeting 2	*N* = 14 *n* = 7 PAs (5 cancer survivors, 2 caregivers) *n* = 4 PERC personnel *n* = 3 AstraZeneca personnel	When in your journey would you be open to hearing about general clinical research? When in your treatment plan would have been optimal to hear about a specific clinical trial? How could this treatment study be presented that it would not feel like a “last ditch”? How could friends and family be brought into the conversation [surrounding clinical research]?	General: From a patient perspective, what are the best timing and delivery of clinical trial education? Specific: What do patients want to hear, when they want to hear it, what is important to them regarding clinical trial participation? What are the best practices for framing a clinical trial as a treatment option?	
Meeting 3	*N* = 13 *n* = 7 PAs (5 cancer survivors, 2 caregivers) *n* = 5 PERC personnel *n* = 2 AstraZeneca personnel	Reviewed synthesized outputs from Meetings 1 and 2	General: Provide patients a summary of potential actions based on their feedback. Specific: Demonstrate patients as partners in this collaboration. Provide confirmation that the “patient’s voice” is being heard and considered.	

Note PERC = Patient Engaged Research Center; PA = Patient Advisor

Each meeting lasted approximately 60–90 min. Equivalence is primarily an issue when multiple moderators are used in qualitative research, such that moderator experience and interviewing style may impact the content and flow of the group discussions [14]. Thus, to ensure rigor in this qualitative inquiry, consistency of moderators was taken into consideration. Throughout meetings, facilitators would continually summarize the narrative evolving, confirming that accurate information (in terms of content, sentiment, and valence) was being recorded. All meetings were conducted in English. For the document review process, the field notes kept by both HFH and industry stakeholders (i.e., PERC and AstraZeneca personnel, respectively), which included responses to the discussion prompts, were transcribed verbatim and shared with all authors.

### Data analysis

*Formation phase.* Based on the Collaborative Process Factors found in the Formation phase of the Model of Research-Community Partnership [10, 15], some members of the CAP (i.e., PERC and AstraZeneca personnel) informally discussed which facilitating and/or hindering factors were present in the partnership development. These members of the CAP were asked to think retrospectively. The categories of ‘interpersonal’ (i.e., quality of relationships or communication among CAP members) [10] and ‘operational’ (i.e., logistics and quality of partnership functioning) [10] processes were used to describe the representative process factors identified by PERC and AstraZeneca personnel.

*Execution of Activities phase.* Based on Braun and Clarke’s [16] recommendations, six phases were implemented during the thematic analysis of the document review (i.e., field notes from the partnership synergy and knowledge exchange meetings): [1] familiarizing yourself with your data [2], generating initial codes [3], searching for themes [4], reviewing themes [5], defining and naming themes, and [6] producing the report. An industry stakeholder (e.g., an AstraZeneca personnel), who had been present at all three meetings, and a HFH qualitative researcher (team member on PERC and lead author) became familiar with the data set by assigning individuals codes, transcribing the field notes and reading the transcripts. Specifically, based on the exploratory nature of our topic and the desire to approach this research using a patient-centered approach, a data-driven inductive approach was used. This type of inductive analysis involves developing codes that are emergent, meaning that they were concepts, actions, or meanings, that evolved from the data [17, 18]. From here, themes were developed through several iterations of interaction with the text and codes. During this interpretive phase of the data analysis three overarching themes (i.e., trials as a treatment option, leaving a legacy, timing is critical) were identified that were felt to capture the phenomenon described in the raw data.

The three overarching themes (i.e., trials as a treatment option, leaving a legacy, timing is critical) were first presented to all PERC and AstraZeneca personnel. A negotiated approach amongst PERC and AstraZeneca personnel was used to finalize the themes and subsequent textual components, a process where after coding of the text, active discussion is used to arrive at a final version in which most, if not all, coded messages have been brought into alignment [19]. Next, during the third Partnership Synergy and Knowledge Exchange meeting (see Table 1), themes were discussed openly amongst all members of the CAP (including PAs of the Cancer Center and Head and Neck Cancer PFAC). Feedback was obtained and it was felt that the three overarching themes (i.e., trials as a treatment option, leaving a legacy, timing is critical) were representative.

## Results

Members of the CAP, retrospectively, identified seven (out of 12) facilitating factors and three (out of 13) hindering factors present during the Formation phase. Using a document review process, field notes from the three meetings were analyzed using an inductive qualitative approach and three major themes emerged. The results are broken down further for both the Formation phase and Executions of Activities phase below.

### Formation phase

Five of the facilitating factors identified were in the interpersonal category and two were in the operational category. Two of the hindering factors identified were in the interpersonal category and one was in the operational category. Some members of the CAP provided examples for justification behind choosing the presence of certain factor. See Table 2 for a full description of the Formation phase results.

**Table 2:** Retrospectively identified facilitating and hindering factors during a community-academic partnership Formation phase categorized by collaborate process factors

Factor	Definition	Category*	Evidence from the CAP
*Facilitating factors* (*n* = 7)			
Shared vision, goals, and/or mission	• Partners share the same identified vision or values. • Partners identify the same goals or mission for CAP.	Interpersonal	• All partners value involving patients in research and decisions; ensuring their voices are heard • The length of time the CAP has existed and the willingness to continue to collaborate to do research
Effective and/or frequent communication	• Partners engage in ongoing communication that is open and respectful. • Communication that encompasses personal and professional matters.	Interpersonal	• Consistent follow ups, check ins, and development of next steps • AstraZeneca would do cycles of action and reflection and bring that process to meetings for dissemination
Trust between partners	• Partners have faith in the honesty, integrity, reliability, and/or competence of one another. • Partners are comfortable sharing because they believe that the sensitive information that they provide in the collaboration will remain in the group.	Interpersonal	• CAP members do introductions • PERC emphasizes storytelling and creating a safe space, thus building trust through vulnerability • From inception • AstraZeneca shared relevancy of research, their roles; creates a sense of investment
Respect among partners	• Partners honor and value one another’s opinions. • Partners are careful to ensure that each member is able to share his or her beliefs.	Interpersonal	• Patient Advisors are asked if they are interested in CAP involvement; invitation and acceptance of that invitation, creating agency and respect
Good relationship between partners	• Partners work well together, group cohesion, strong reciprocal relationship, get along well, or like each other.	Interpersonal	• Demonstrated through the long-standing relationship of the CAP
Good initial selection of partners	• Selecting the “right” people to be a part of the collaborative group. • The personality characteristics of partners contribute to the success of the CAP.	Operational	• Those who had experience in cancer care were asked to participate • Past success of CAP members in research endeavors
Mutual benefit for all partners	• All partners benefit from the group’s progress. • Benefit may be different, but all receive some benefit.	Operational	• PERC and opportunity to engage and give voice to patients • AstraZeneca use patient’s voice to make impact • Patient Advisors feel heard and are making an impact
*Hindering factors* (*n* = 3)			
Poor communication among partners	• CAP has limited or unclear methods of communication. • Partners experience difficulty maintaining communication.	Operational	• More guidance needed on timeframes and wait periods from meeting discussions to tangible actions • AstraZeneca to be clearer on who (i.e., chain of command) incorporates CAP information and research outcomes to be integrated into model for patient engagement
Inconsistent partner participation or membership	• There is inconsistent or fluctuating partner attendance at meetings. • CAP membership is inconsistent. There is attrition or turnover in partnering agencies/organizations or individuals.	Interpersonal	• Started discussion pre-COVID and then had to adapt; moved to digital meetings • Normally better participation face to face; technology challenges • Some CAP members are head and neck cancer patients and digital environment created physical limitations
Differing expectations of partners	• Struggles emerge because not all members expect the same structure, procedures, and/or outcomes.	Operational	• Patient Advisors wanted to see action and implementation with more immediacy, which was not feasible for AstraZeneca

*Note* Factors and definitions are taken from Gomez et al., (2018). Based on Gomez et al. [10]. , lack of mutual benefit and lack of community impact do not appear as hindering factors in the article by Drahota et al. [9]. , but were derived from additional literature [15, 20, 21] and included in the current study. CAP = Community-Academic Partnership.*Category is based on the Collaborative Process Factors found in the formation phase of the Model of Research-Community Partnership [10, 15]

### Executions of activities phase

In the subsequent paragraphs, each of the three themes identified during the interpretive phase of the data analysis are discussed and sample quotes, identified by participant IDs, are provided.

#### Trials as a treatment option

Participants noted that often clinical trials are incorrectly perceived, and as a treatment option can feel like a desperate last resort, “I always viewed clinical trials as “last resorts”. It was a welcome discovery to learn that they are viable options for first line treatment” (Participant 2). Although clinical trials are offered in first line therapy, participants agreed that, from their perspective, the concept of clinical trial participation was not discussed in their general healthcare. Therefore, when research is only discussed once they have been diagnosed it feels too overwhelming and a negative connotation may emerge: “Can take a while to establish comfort level with clinical trials. Had to do with how option was presented. Has to be messaged properly. Physician initially approached it as ‘this is a last-ditch effort’ and came across as negative” (Participant 4) and “General perception [of clinical trials] is last ditch. To avoid that perception, as early as possible you [healthcare providers] have to talk to the idea [of clinical trials]” (Participant 1).

Participants reflected on how the COVID-19 pandemic has raised awareness of clinical trials. COVID-19 research may change perceptions of clinical trials in that it “has introduced clinical research (how to develop a vaccine) and stages of clinical research” (Participant 1) to the general public. As some participants noted clinical trial participation as a treatment option is not always “in the mind of the average patient” (Participant 1). The serge of media attention that the COVID-19 vaccine trials have gained could assist in leveraging participation in this type of research in the future because patients may feel more educated on the process.

#### Leaving a legacy

Amongst participants, a narrative developed around how a “clinical trial is a way for the patient to give back and have a legacy” (Participant 5). For example, a participant mentioned that “even if it might not work out the best for you, it will for someone else and that’s part of the process [of clinical trials]” (Participant 3). To build participation in clinical trials and thus their “legacy” it was imperative for patients to understand the “why” for participating in this type of research. The education around how the clinical trial is introduced or explained was critical. As mentioned by one participant, “it’s all in the presentation of the information” (Participant 7). This true understanding of the goals and benefits of the trial gives patients a sense of purpose.

Discussion ensued on how participation could generate positive feelings, create additional opportunities in clinical trials, and “lay the groundwork for future treatments” (Participant 8). For example, a participant noted “I would be glad to know that I made a positive impact by participating. I am currently participating in a study that does not require active treatment, rather serial monitoring and it feels good to know that even that little contribution may eventually make a positive impact on someone’s life” (Participant 2). Participants emphasized the need for storytelling, sharing of other patients’ success and the beneficial impacts of being involved in a clinical trial; “it’s important to tell stories about the impact [of clinical trials]” (Participant 5). Providing this context could help leverage participation from others. In addition, the pharmaceutical/biotech industry sponsors of the clinical trials need to be framed in a way that evokes a relationship with the patients, “as a partnership for the good of humanity” (Participant 5), and in a way that makes a patient want to be involved in the research so that they too can leave a legacy.

#### Timing is critical

All participants agreed that: “timing is everything to approach a patient about a clinical trial” (Participant 6). Consensus amongst participants was that earlier education of clinical trial opportunities for patients and informative presentations by providers would be best, with one participant stating they “wished it [clinical trial] was offered earlier” (Participant 3). With a participant suggesting that, during the time of diagnosis it would be helpful “to know viable alternatives which should include clinical trials” as they believed, “There is never too much information for a newly diagnosed cancer patient. The patient just needs to feel comfortable making the decision.” (Participant 4).

However, one participant noted that early on in care: “You’re overwhelmed and focused on your treatment. Be careful about when you discuss this option with the patient because they don’t need another ‘thing’ to manage. May be better received further into treatment” (Participant 6). In turn, this comment developed discussion around that perhaps it is more imperative that the provider or individual presenting the clinical trial option have a strong awareness of *if* the patient and their caregiver, are “able” to receive information surrounding it as an option: “Sooner is better, but the challenge then is patient and caregiver - are they able to hear it?” (Participant 5).

## Discussion

Guided by the Model of Research-Community Partnership [10] the purpose of this study was to use a CAP to better understand what facilitates and/or hinders participation in clinical trials as a treatment option, among a diverse patient population. Overall, the Formation and Execution of Activities phases are highlighted, evaluated, and analyzed. This study contributes to research and practice by systematically reporting the CAP’s characteristics, highlighting the longitudinal relationships, and describing the interpersonal and operational processes that have facilitated and/or hindered this collaborative effort. Moreover, the current study provides valuable patient-centered perspectives on barriers and challenges of clinical trials as an accepted treatment option and how to improve the perception of clinical trial participation. Similar to previous CAP literature [22, 23], the discussion has been framed using some of the choice points for quality in action research, including *partnership and participation, actionability, reflexivity*, and *significance* [24].

*Partnership and participation* refers to understanding the quality of relationships formed with stakeholders and their involvement in inquiry [25] and the extent of participation and the relational component of research being referred to [24]. In the current study this can be described specifically within the systematic report of the CAP’s characteristics and the Collaborative Process Factors identified in the Formation phase. Similar to previous literature [10] interpersonal processes, compared to operational processes were referenced as influential facilitating factors more often during the CAP’s Formation phase. These interpersonal processes included a shared mission and vision, effective communication, trust and respect between CAP partners which led to a good working relationship of the CAP. Interpersonal factors, similar to those identified as facilitating factors in the current study, have been noted throughout the literature as important elements of a CAP’s success [10, 15, 23, 26–28]. Having a shared group mission and vision has been cited in previous CAP research as of higher importance over other interpersonal processes during the Formation phase [10, 23, 28]. Thus, taken together, future action research projects or CAPs should seek partners that have identified and share the same vision and values. Moreover, for those CAPs involving patients, the goals or mission surrounding the need for the patient-perspective should be emphasized in the partnership to further facilitate collaborative development.

This study being *actionable* refers to the extent to which it provides new ideas that guide action in response to need [24, 25]. Previous research indicated a need to better understand the challenges and barriers when it comes to recruitment and retention in clinical trial research [1–3]. Moreover, anecdotally, based on interaction with the CAP, as well as various healthcare providers members, a need was expressed to better integrate a patient-perspective approach to drive clinical trial participation, rather than relying on assumptions or quantitative data. Within the current study, the finding that the interpersonal process of having a shared group vision was one of the most influential facilitating factors during the CAP’s development indicates that a mutual need existed. The Partnership Synergy and Knowledge Exchange meetings during the Execution of Activities phase resulted in possible implications and potential actions, which were derived from the three coded themes in the data. There is a need to change perception to clinical trials as a treatment option. How and when a physician presents trials as well as how patients learn about trials is important in developing the patient’s perception. Results of the current study suggest a need to clearly communicate the short- and long-term as well as direct and indirect benefits of clinical trial participation. For patients who participate on trials, there is a need to share their results and impact, a need to work directly with other patients to tell their stories. Findings help provide new ideas for future research such as patient friendly summaries of trial results communicated to patients who participate and integrating real patients into educational materials.

As a way for CAP members to understand and acknowledge their role as instruments of change among stakeholders [24, 25], *reflexivity* was used throughout the project. Overall, as the Formation phase suggest facilitating factors such as shared vision, effective and/or frequent communication, and mutual benefit for all partners were present, this acknowledges the intention of reflexivity by the CAP. As outlined in the methodology, AstraZeneca’s stakeholders would reflect after the Partnership Synergy and Knowledge Exchange meetings during the Execution of Activities phase, refine their discussion points/focus, summarize research findings, and integrate the participant perspectives. AstraZeneca would use this reflexive activity to develop discussion points/focus and goals to deliver back to the CAP members at the subsequent meetings. AstraZeneca’s core values include, putting patients first, following the science and doing the right thing. Moreover, AstraZeneca wants to ensure that patients are first in mind when developing and managing clinical research. As such, the tone of conversation with patient stakeholders is always one of respect and appreciation for their time. AstraZeneca ensured that at the beginning of conversations they provided an update on any outstanding work or activities that were being moved forward due to group feedback. AstraZeneca ensured that meetings were closed with a recognition of the time and energy PAs provided and that their ongoing participation was allowing improvements for all potential patients. AstraZeneca and PERC team members (the authors) strive to hold themselves and others accountable for making decisions in the best interests of patients. We (the authors) think of patients as people and tailor solutions and approaches to meet their needs. We seek to understand the healthcare environment and external trends. We seek partners and collaborators that share our passion for science. We do the right thing and act with integrity, even when it is difficult. During this experience we made sure to bring forward the various voices of the CAP to ensure an equitable process. When working with the Cancer Center and Head and Neck Cancer PFACs, we summarized their comments and provided them with a presentation of what we heard from them (see Data Analysis - Executions of Activities Phase). We allowed the PAs to provide consultative guidance, comment and clarify any statements, provide feedback on the themes generated, and provided a direct contact to reach out for assistance or support. In addition, investigating the findings from the current study allows for reflexivity moving forward into the next stages of these collaborative efforts, and will aid in continuing to foster a positive and productive CAP.

Defined as having meaning and relevance beyond an immediate context [25], *significance*, is demonstrated in the current study. The Cancer Center as well as the Head and Neck Cancer PFACs have been provided with the opportunity to be involved in meaningful conversations around clinical trial participation. The themes identified and narratives which ensued in the Execution of Activities phase have aided in next steps of the CAP. Beyond this immediate study, *significance* is demonstrated by the proposed future research projects with the CAP which have been developed based on the discussions in the Partnership Synergy and Knowledge Exchange meetings. For example, getting feedback on patient facing material so that messaging and information surrounding clinical trials will be perceived with a positive undertone (vs. a last-ditch effort) is under review for feasibility of implementation. Moreover, future research with the CAP will aim to evolve beyond a patient-centered design and into a co-design approach. The current study has utilized a patient-centered design [29–31], in that the team strived to ensure that the needs of the patient are centermost, however, the project was not identified by patients. In a co-design approach patients help identify the process or project that needs to be designed (or redesigned) based on their personal experience [32], which in essence, are the themes that have been identified through the qualitative analysis of the Execution of Activities phase.

Several limitations of the current study need to be acknowledged. Only perspectives of members in a single CAP, are represented here, which may limit generalization of findings. Exploring additional CAPs is needed where collaborative, patient-centered efforts involving multiple stakeholders are occurring so to help bring richer and more representative perceptions. Within the Formation phase, interpersonal and operational processes were limited to only retrospective reflection from some of the CAP members. To gain a more robust understanding of the CAP development future research could use a mixed method approach to gain both quantitative and qualitative data. Moreover, to decrease the researcher-driven study design, a co-design approach [31] and Colaizzi’s descriptive phenomenological method [33] for data analysis could be used. Although the that data used in this study is not appropriate for Colaizzi’s descriptive phenomenological method, the seventh and final step in Colaizzi’s method, involves gaining the participants’ input on the phenomena developed from the themes identified through the data coding process [33]. Although this method has been criticized in that the researcher and participant inevitably have different perspectives [33], within a CAP, harmonizing final aspects of the data analysis could allow for further insight into how their role makes them an instrument of change in addition to addressing the hindrance of differing expectations of partners. In addition, using this method and approach would have better provided the PAs and opportunity to fit the criteria the International Committee of Medical Journal Editors’ (ICMJE) recommends for authorship [34]. Based of the current study design, in which the PAs involvement was limited to consultative guidance, acknowledgement is more appropriate [35]. Despite these limitations, the current study has important strengths including giving a detailed account of the Formation phase and providing valuable patient-centered perspectives on barriers and challenges of clinical research as an accepted treatment option and how to improve the perception of clinical research.

## Conclusions

To better understand and address barriers and challenges of clinical research as an accepted treatment option, this article describes the Formation and Execution of Activities phase within a CAP that brought upon a collaborative effort between industry stakeholders, a healthcare/academic institution, and patients/families with lived experiences as cancer survivors and/or caregivers. This study contributes to research and practice by systematically reporting the CAP’s characteristics, highlighting the longitudinal relationships, and describing the influential interpersonal and operational processes present in the Formation phase. Guided by the Model of Research-Community Partnership [10], the facilitating and hindering factors were explored retrospectively. Both interpersonal and operational factors were mentioned. Interpersonal factors expressed as facilitators more often, compared to operational factors expressed as hinderances more often. Similar to other action research a shared group mission and vision was highlighted as an important facilitator for a CAPs development. The Partnership Synergy and Knowledge Exchange meetings during the Execution of Activities phase resulted in possible implications and potential actions, which were derived from the three coded themes in the data. Overall, this study helps to provide a patient-centered perspective on barriers and challenges of clinical trial participation as an accepted treatment option and how to improve the perception of future clinical trials.

## Data Availability

Not applicable.

## Abbreviations

CAP: Community Academic Partnership
HFH: Henry Ford Health
PERC: Patient Engaged Research Center
PA: Patient Advisor
PFAC: Patient Family Advisory Council
PCORI: Patient Centered Outcomes Research Institute
